# Supplementary material for: Transcriptomic analysis reveals that pyruvate kinase potentially plays a key role in the differentiation of Spirometra mansoni proglottids by regulating the glycolysis pathway
Source: PLoS Negl Trop Dis. 2025 Oct 9;19(10):e0013570. doi: 10.1371/journal.pntd.0013570 (PMC12510601; doi:10.1371/journal.pntd.0013570)
Supplement: S2 Fig — (PDF) [file pntd.0013570.s015.pdf]

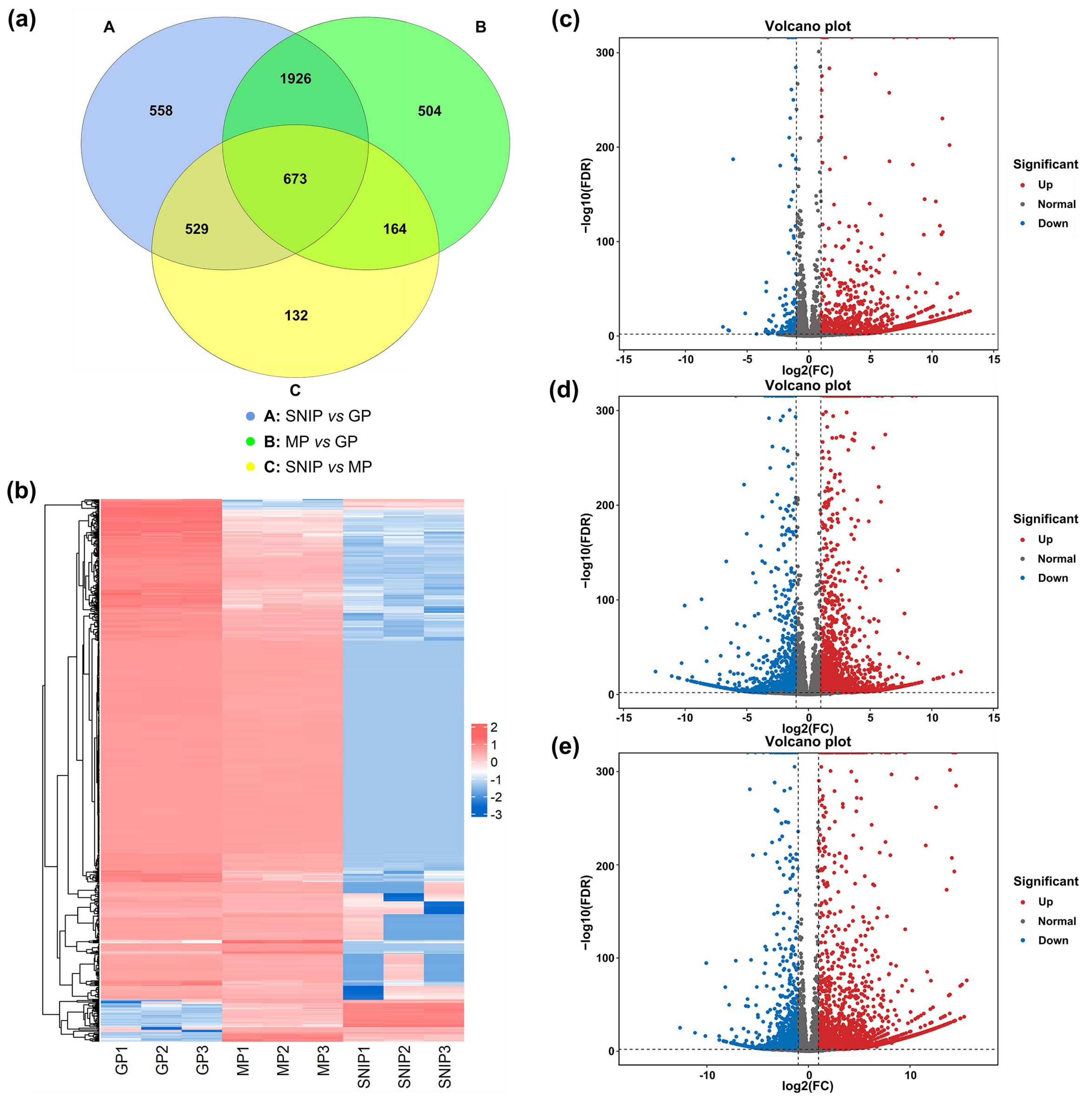

S2 Fig Screening of differentially expressed genes among different segments of *S. mansoni*. (a) Venn diagram of all DEGs. (b) Heatmap of all DEGs. Red and blue indicate the DEGs that were significantly upregulated and downregulated, respectively, and white indicates the DEGs for which the change in expression level was not statistically significant. (c), (d), and (e) Volcano plots of DEGs between SNIPs and MPs, MPs and GPs, and SNIPs and GPs, respectively. The red dots represent significantly upregulated genes, the blue dots represent significantly downregulated genes, and the grey dots represent genes with no statistically significant change in expression levels.
