# Supplementary material for: Transcriptomic analysis reveals that pyruvate kinase potentially plays a key role in the differentiation of Spirometra mansoni proglottids by regulating the glycolysis pathway
Source: PLoS Negl Trop Dis. 2025 Oct 9;19(10):e0013570. doi: 10.1371/journal.pntd.0013570 (PMC12510601; doi:10.1371/journal.pntd.0013570)
Supplement: S3 Fig — (PDF) [file pntd.0013570.s016.pdf]

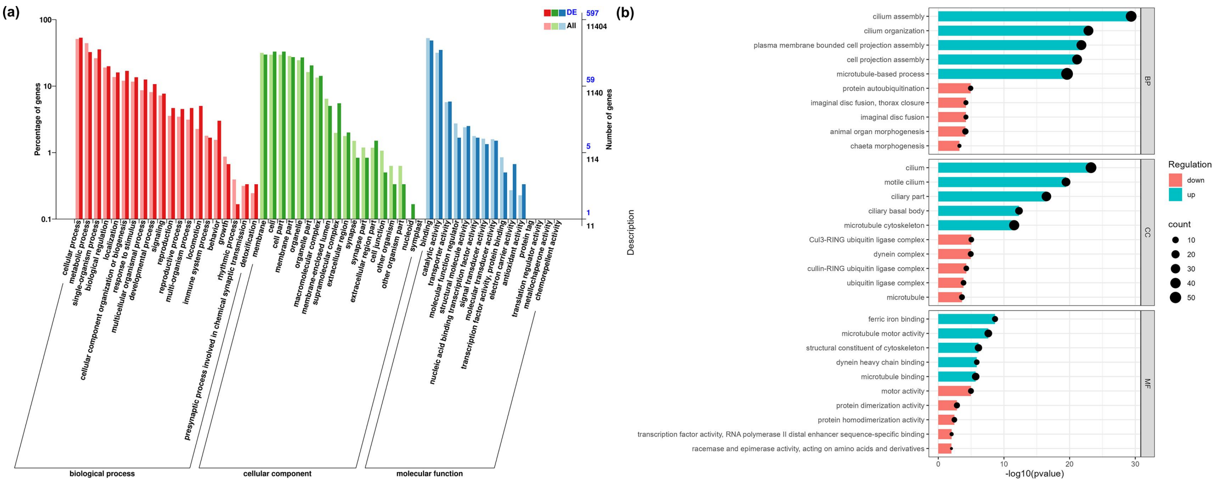

S3 Fig GO analysis of DEGs between SNIPs and MPs. (a) GO subcategories of DEGs. (b) Top 5 enriched terms in the BP, CC, and MF categories for upregulated genes in SNIPs (red) and MPs (green).
