# Supplementary material for: Transcriptomic analysis reveals that pyruvate kinase potentially plays a key role in the differentiation of Spirometra mansoni proglottids by regulating the glycolysis pathway
Source: PLoS Negl Trop Dis. 2025 Oct 9;19(10):e0013570. doi: 10.1371/journal.pntd.0013570 (PMC12510601; doi:10.1371/journal.pntd.0013570)
Supplement: S8 Fig — (PDF) [file pntd.0013570.s021.pdf]

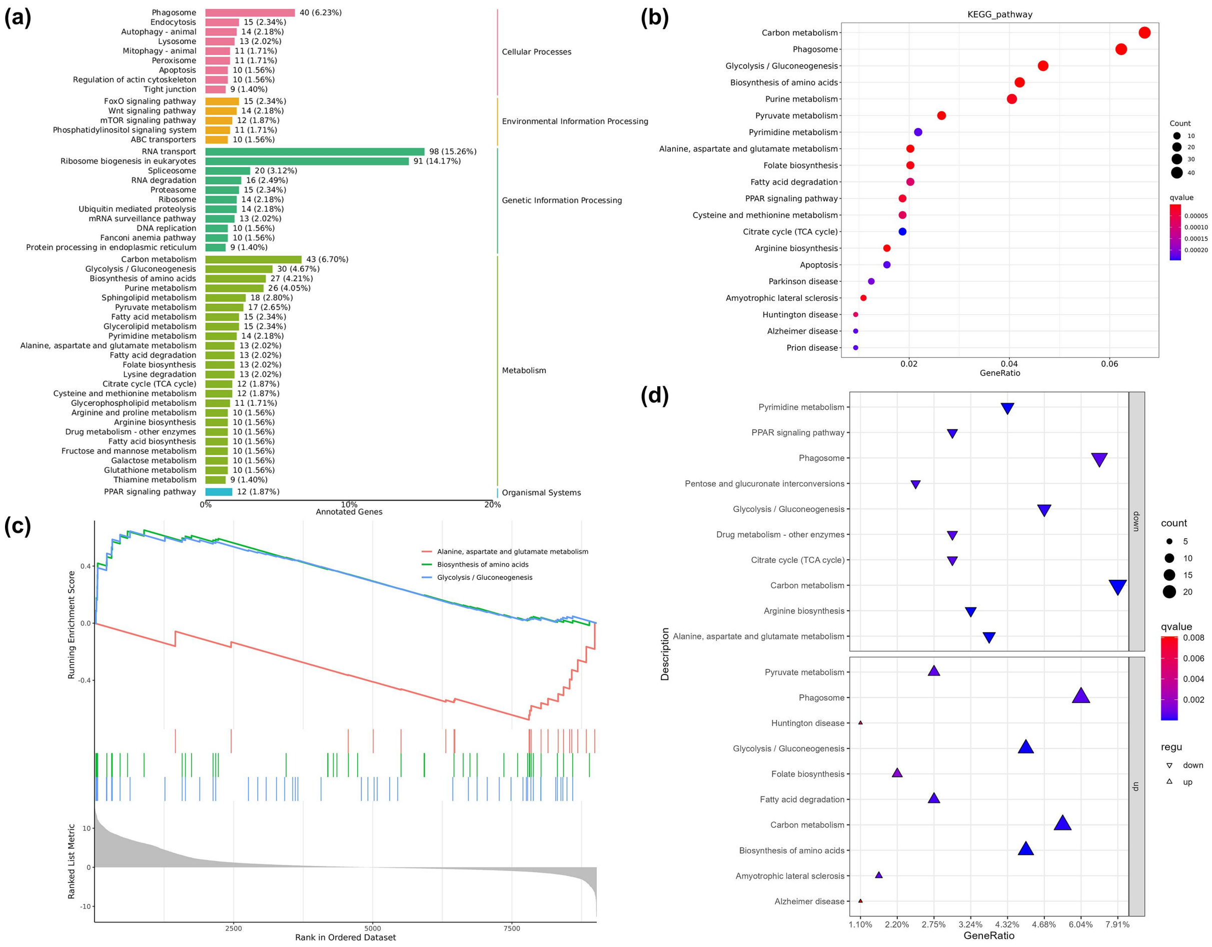

S8 Fig KEGG analysis of DEGs between SNIPs and GPs. (a) KEGG classification of DEGs. (b) Top 20 enriched KEGG pathways of DEGs. (c) GSEA enrichment plot of pathways significantly enriched with DEGs. (d) Top 10 KEGG enriched pathways with significantly upregulated DEGs in SNIPs (▽) and GPs (△).
