# Supplementary figures and images for: Transcriptomic analysis reveals that pyruvate kinase potentially plays a key role in the differentiation of Spirometra mansoni proglottids by regulating the glycolysis pathway
Source: PLoS Negl Trop Dis. 2025 Oct 9;19(10):e0013570. doi: 10.1371/journal.pntd.0013570 (PMC12510601; doi:10.1371/journal.pntd.0013570)

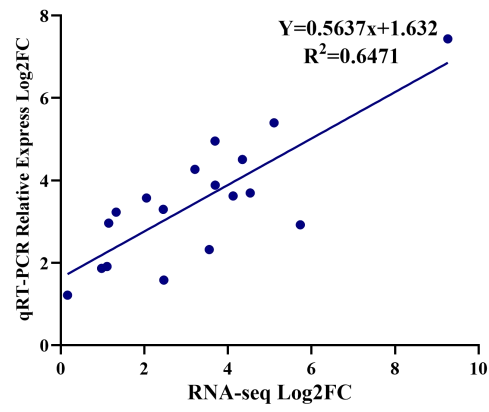

**S10 Fig** Correlation of the expression levels of DEGs between the RNA-seq and qRT-PCR results.

Supplement: S10 Fig — (PDF) [file pntd.0013570.s023.pdf]
