# Supplementary material for: Transcriptomic analysis reveals that pyruvate kinase potentially plays a key role in the differentiation of Spirometra mansoni proglottids by regulating the glycolysis pathway
Source: PLoS Negl Trop Dis. 2025 Oct 9;19(10):e0013570. doi: 10.1371/journal.pntd.0013570 (PMC12510601; doi:10.1371/journal.pntd.0013570)
Supplement: S11 Fig — (PDF) [file pntd.0013570.s024.pdf]

(a)

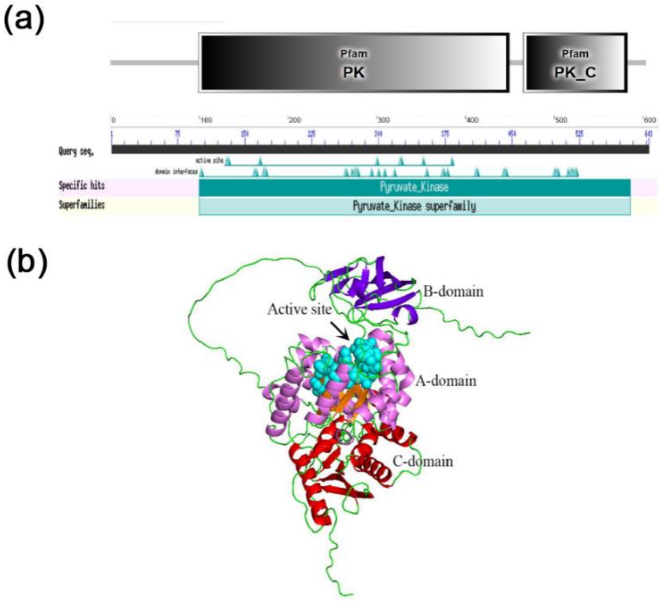

S11 Fig Characterization of the basic physicochemical properties of *Sm*PK1. (a) Functional domain prediction. (b) Tertiary structure of the *Sm*PK monomer: the blue spheres represent active sites; the pink α-helices and orange β-sheets constitute domain A; the purple structure indicates domain B; and the red structure denotes domain C.
