# Supplementary material for: Transcriptomic analysis reveals that pyruvate kinase potentially plays a key role in the differentiation of Spirometra mansoni proglottids by regulating the glycolysis pathway
Source: PLoS Negl Trop Dis. 2025 Oct 9;19(10):e0013570. doi: 10.1371/journal.pntd.0013570 (PMC12510601; doi:10.1371/journal.pntd.0013570)
Supplement: S9 Fig — (PDF) [file pntd.0013570.s022.pdf]

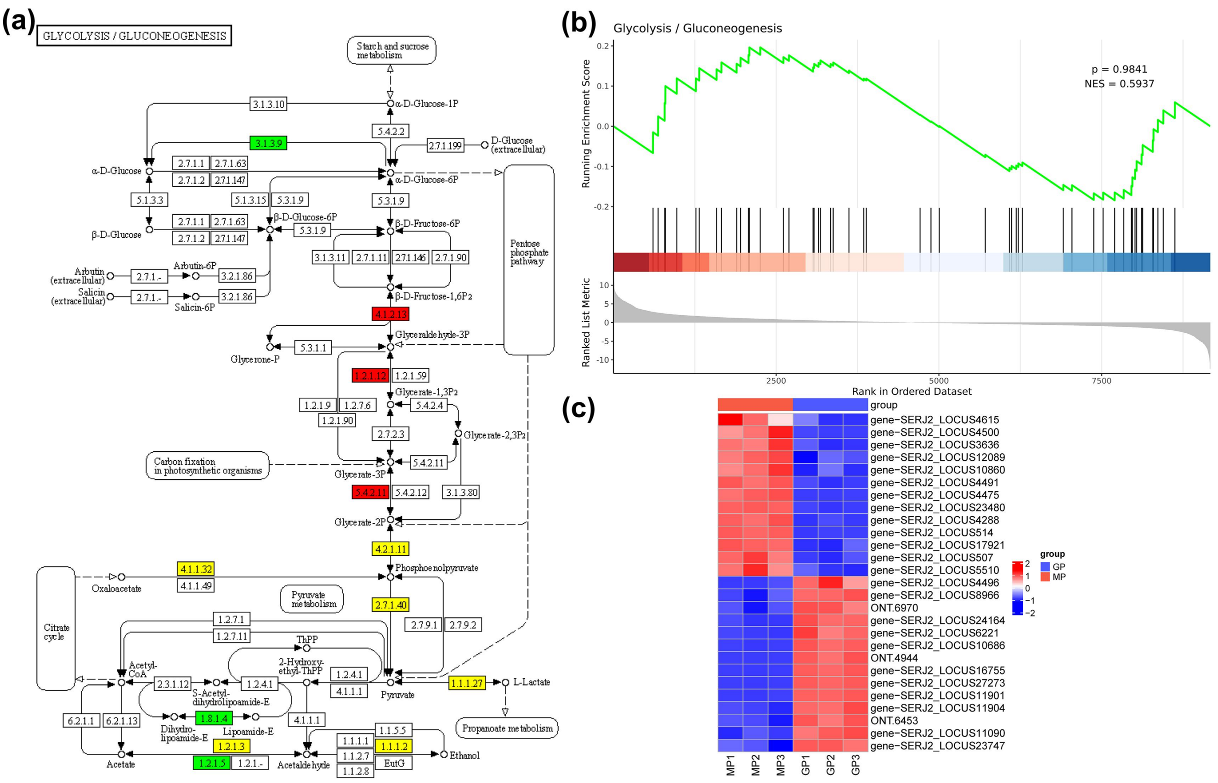

S9 Fig Analysis of the glycolysis pathway in MPs and GPs. (a) Twenty-seven DEGs involved in the glycolytic pathway were identified in the MPs and GPs. The numbers in boxes represent genes. Red and green symbols represent genes upregulated in GPs and MPs, correspondingly. Yellow symbols represent genes with mixed regulation. (b) Gene set enrichment analysis of the glycolysis pathway in MPs and GPs. A positive NES indicates significant enrichment of this pathway in GPs. (c) Heatmap of 27 DEGs in the glycolysis pathway.
