# Supplementary material for: Transcriptomic analysis reveals that pyruvate kinase potentially plays a key role in the differentiation of Spirometra mansoni proglottids by regulating the glycolysis pathway
Source: PLoS Negl Trop Dis. 2025 Oct 9;19(10):e0013570. doi: 10.1371/journal.pntd.0013570 (PMC12510601; doi:10.1371/journal.pntd.0013570)
Supplement: Attachment — Submitted filename: Response to comments.docx [file pntd.0013570.s026.docx]

**Dear Editors:**

Thank you for your letter and the reviewers’ comments on our manuscript entitled “**Transcriptomic analysis reveals that pyruvate kinase plays an important role in the differentiation of *Spirometra mansoni* proglottids by regulating the glycolysis/gluconeogenesis pathway (PNTD-D-25-00822)**”. These comments are all valuable and very helpful for revising and improving our paper. We have studied the comments carefully and have revised our manuscript. We hope that the revised MS will meet your requirements. The main revisions are included in the manuscript, and the responses to the reviewers’ comments are detailed below.

**Responses to the reviewer’s comments**

**Q1.** The authors should carefully consider the reviewer concerns, including sharing data indicating the data underlying the genotyping of S. mansoni. As pointed out by reviewer 2, based on the link provided, it seems that the S. erinaceieuropaei genome reference was used for the analysis, but this is not acknowledged in the text except for in the link provided. If a different species or reference was used then this needs to be clarified, since it affects all downstream results. It is not appropriate to use a different species assembly to do a gene-level analysis since different species may have substantial expansions and contractions of gene families.

**Response:** Thank you for your suggestion. Although the reference genome used in this study is that of *Spirometra erinaceieuropaei* (GCA_902702965.1) (Kikuchi et al., 2021), recent taxonomic studies have revealed that most *Spirometra* isolates from Asia have been misidentified (Kuchta et al., 2021). Specifically, the Asian isolates previously identified as *S. erinaceieuropaei* should actually be classified as *S. mansoni*, whereas the true *S. erinaceieuropaei* is distributed only in certain areas of Europe. Consequently, the parasite strain employed in this study belongs to the same species as the one used for the reference genome. Nevertheless, to maintain consistency with the original literature, we continue to use the name *S. erinaceieuropaei* for the reference genome in our study.

Kikuchi T, Dayi M, Hunt VL, Ishiwata K, Toyoda A, Kounosu A, Sun S, Maeda Y, Kondo Y, de Noya BA, Noya O, Kojima S, Kuramochi T, Maruyama H. Genome of the fatal tapeworm Sparganum proliferum uncovers mechanisms for cryptic life cycle and aberrant larval proliferation. Commun Biol. 2021;4(1):649.

Kuchta R, Kołodziej-Sobocińska M, Brabec J, Młocicki D, Sałamatin R, Scholz T. Sparganosis (Spirometra) in Europe in the Molecular Era. Clin Infect Dis. 2021; 72: 882-890.

**Q2.** Also as mentioned by reviewers, this is not a chromosomal-level assembly so the chromosome analysis in Figure 6 should be also be clarified or changed to scaffolds matching the assembly numbers. The mapping rates are also very high for a fragmented draft assembly to the wrong species, so it's unclear what was done. In a previous study (PMID:36406076), the submitting research group cited the *S. erinaceieuropaei* genome paper after saying that the *S. mansoni* genome has been published, so perhaps they consider these to be the same species or that the genome is incorrectly labeled?

**Response:** Thank you for your comment. As mentioned above, the *Spirometra erinaceieuropaei* genome referenced in this study is actually the genome of *Spirometra mansoni*. According to your suggestion, we have made the following revisions to Figure 6: 1) Terminology correction: "Chromosomal localization" in Figure 6A has been replaced with "scaffold-level localization". All relevant text has been updated by changing "chromosomes" to "scaffolds". 2) Method modification: The original description "To precisely map *Sm*PK genes on chromosomes, distribution information was collected from the *S. mansoni* genome, and gene distribution maps were generated using TBtools2 software" has been revised to "To precisely map *Sm*PK genes on scaffolds, distribution information was collected from the *S. erinaceieuropaei* draft genome (NCBI accession GCA_902702965.1), and gene distribution maps were generated using TBtools2 (v2.136) software. ". 3) Figure legend modification: The original legend "Figure 6A. Chromosomal localization: the green columns represent different chromosomes of *S. mansoni*" has been revised to " Scaffold-level localization: the green columns represent different scaffolds of *S. erinaceieuropaei* (GCA_902702965.1)".

**Q3.** Importantly, raw sequence data for each sample must be uploaded to a repository in order to comply with PLOS NTD Data availability requirements (https://journals.plos.org/plosntds/s/data-availability). Given the apparent confusion with much of the analysis, in order to make the data reproducible, supporting information tables with differential expression statistics for each gene in each comparison would also help to facilitate the usability of the data for other researchers.

**Response:** Thank you for your suggestion. All the original data used in this study have been uploaded to a public database as follows: The RNA-seq data from this study have been deposited in the NCBI Sequence Read Archive (SRA accession number PRJNA1248218) and are accessible through accession numbers SAMN47853541, SAMN47853542, SAMN47853543, SAMN47853544, SAMN47853545, SAMN47853546, SAMN47853547, SAMN47853548, and SAMN47853549. In addition, in the comparative analysis, the statistical data of the differential expression of each gene have been added to the supplementary materials (Supplementary Tables S5–S8).

**Q4.** Reviewer 1 provided a reference suggesting that gluconeogenesis is not present in tapeworms or other parasitic flatworms. I wasn’t able to access a copy of the book referenced to confirm this, but please perform some additional research to check on this. The text could be revised to just say glycolysis with no emphasis on gluconeogenesis, without any substantial changes.

**Response:** Thank you very much for your suggestion. Regarding the comment from Reviewer 1, we have also searched the relevant literature, but unfortunately, we have not found any related evidence. According to your suggestion, we have revised the text, focusing only on the glycolytic pathway and no longer emphasizing the gluconeogenesis pathway.

**Q5.** Also, please ensure that statistical approaches are sufficiently described, including in figure captions.

**Response:** Thank you for your comment. We have carefully checked the parts of the manuscript that involve statistical methods, including the figure legends and table captions, and have supplemented and improved them.

**Reviewer #1:**

**Methods**

**Q1** Are the objectives of the study clearly articulated with a clear testable hypothesis stated? Not entirely.

**Response:** We have revised the research objectives in accordance with your suggestions to make them clearer and more explicit.

**Q2** Is the study design appropriate to address the stated objectives? Not entirely.

**Response:** We have made revisions according to your suggestions in the MS.

**Q3** Is the population clearly described and appropriate for the hypothesis being tested? Not in all the experiments.

**Response:** We have checked all the testing methods to ensure their accuracy and correctness.

**Q4** Is the sample size sufficient to ensure adequate power to address the hypothesis being tested? Not in all the experiments.

**Response:** We have made revisions in line with your suggestions.

**Q5** Were correct statistical analysis used to support conclusions? Not in all the experiments.

**Response:** We have checked all the statistical methods to ensure their accuracy and correctness.

**Conclusions**

**Q6** Are the limitations of analysis clearly described? No, they are not clearly described.

**Response:** According to your suggestions, we have supplemented and elaborated on the limitations of the analysis.

**Q7** Do the authors discuss how these data can be helpful to advance our understanding of the topic under study? It is discussed briefly, but the discussion is far from complete for the field.

**Response:** Following your suggestions, we have made further revisions to the discussion section.

**Overview**

**Q8** The manuscript by Zhou and collaborators "Transcriptomic analysis reveals that pyruvate kinase plays an important role in the differentiation of *Spirometra mansoni* proglottids by regulating the glycolysis/gluconeogenesis pathway"presents an interesting work that studies the differentiation of proglottids in the cestode *Spirometra mansoni* by transcriptomics. While the study is clearly presented and the figures and tables are well prepared, it lacks a deep biological interpretation of features relevant to cestode physiology.

**Response:** Thank you very much for your affirmation of this study. Regarding the issue of "in-depth interpretation of tapeworm physiology-related characteristics", we have added the following relevant content in the revised manuscript: "Adult *S. mansoni* show characteristic body differentiation into the scolex, neck and strobila. The neck section is a very small part that occurs after the scolex section, and its function is to continuously bud off the segments. The strobila segments can be divided into immature proglottids (IPs), mature proglottids (MPs) and gravid proglottids (GPs) according to their anteroposterior position and the development of their sexual organs. Just behind the neck are the IPs, and further from the posterior are MPs with mature female and male organs and, finally, those whose uterus contains fertilized eggs. The latter proglottids are described as GPs. The gravid or senile terminal proglottids detach or disintegrate (Conboy, 2009; Bo, et al., 2012)."

Conboy G. Cestodes of dogs and cats in North America. Vet Clin North Am Small Anim Pract. 2009;39(6):1075-90, vi.

Bo X, Zhao W, Zhang H, Kang L, Wang X. Gene expression profiling of in Moniezia expansa at different developmental proglottids using cDNA microarray. Mol Biol Rep. 2012;39(4):4499-507.

**Q9** Gluconeogenesis is not present in tapeworm and other parasitic flatworms (A.G.M. Tielens, J.J. van Hellemond, Unusual aspects of metabolism in flatworm parasites, 2006). This is critical for what the authors show in the work and also in the title. This is an error probably produced by the analysis of the GO terms obtained, these are powerful tools but not always directly useful for tapeworms and other non-classic organisms, where a deeper biological analysis is needed to interpret the results.

**Response:** Thank you for pointing out this critical issue. We fully agree with the importance of this point for the research content and title of the paper. As you noted, this misunderstanding may indeed stem from the direct application of GO terms. Additionally, we have tried to find the important reference you provided; unfortunately, we were unable to locate it. According to your suggestions, we have reexamined the relevant analysis process and comprehensively revised the content related to gluconeogenesis. The specific revisions are as follows: all statements in the paper regarding the existence of gluconeogenesis in tapeworms have been deleted, and both the title and the main text focus solely on the glycolysis process.

**Q10** The final experiment shown in the text is not properly set up. - Lines 262-271 - In vitro” inhibition assay: “Two adult cestodes with similar individual characteristics…” Based on the experimental description, it is difficult to draw reliable conclusions from this part of the study.

**Response:** Thank you very much for pointing out the problems in the design of the in vitro inhibition experiment. This may be due to the lack of clarity in our description. In the revised manuscript, we have made modifications to this issue as follows: Two adult cestodes with similar individual characteristics were sequentially washed three times each with PBS and RPMI 1640 medium to remove contaminants. The adult cestodes were then divided into an experimental group (100 µM tannic acid treatment) and a control group (inhibitor-free incubation). After incubation at 37 °C, 4 uniformly sized GPs were collected from the posterior ends of both the experimental and control cestodes at 3-h intervals (3/6/9 h) and placed into prelabelled centrifuge tubes. Finally, according to the kit instructions, the tissue samples were weighed and processed for subsequent quantification of pyruvate (Sangon Biotech, Shanghai, China), total sugars (Sangon Biotech, Shanghai, China), and triglycerides (Jiancheng Biotech, Nanjing, China). Additionally, the enzyme activity of *Sm*PK1 in GPs was detected using the 2,4-dinitrophenylhydrazine chromogenic method.

**Other points**

**Q11** "medical platyhelminths" is an awkward and non-standard expression, it appears several times in text.

**Response:** Thank you for your suggestion. We have revised the entire manuscript and replaced the expression "medical platyhelminths" with the more standard term "zoonotic cestodes and trematodes".

**Q12** Fig 9. an explanation of why some of the tissues shown are almost identical is required. Example: “Infection serum, Uterus, Alexa 488” and “Anti-SmPK serum, Uterus, Alexa 488” and some others are almost identical, or very similar in structure, why is this the case?

**Response:** Thank you for your question. Regarding this issue, we provide the following explanations. First, the similarity in uterine structure between the infected serum group and the immune serum group stems from the experimental design: uterine tissues from both groups were obtained from consecutive sections of the same mature proglottid (adjacent sections of the same paraffin block), thus having identical morphological features. Additionally, to eliminate technical errors, all processing conditions—including section thickness, antigen retrieval, blocking time, etc.—were strictly standardized, except for the primary antibody type. During imaging, fixed fields of view were used to ensure that images from both groups were captured within the same microscope field whenever possible, minimizing fluorescence intensity discrepancies caused by field deviation. Second, the egg samples were subjected to the same standardized procedures. The similar Alexa 488 fluorescence signals observed in mature and gravid proglottids of both the infected and immune serum groups may be attributed to the unique high-energy metabolic demands of adult cestodes. qPCR quantitative analysis revealed significantly higher expression levels of pyruvate kinase (PK) in the mature and gravid proglottids of adult cestodes than in those of larval cestodes (>2.6-fold, p < 0.01) (Fig. 8B). Given the critical role of PK in glycolysis, this finding suggests that the reproductive phase of adult cestodes (particularly gravid proglottids) may require more active glycolytic metabolism to support energy-intensive physiological activities such as egg production. Consequently, the immune serum group presented stronger PK-specific localization signals (around the uterus and vitelline gland regions), whereas the infected serum group presented more diffuse signals due to the presence of nonspecific antibodies.

**Q13** There are other terms that are awkward in the text, like in Line 431 “significant fluorescence”.

**Response:** Thank you for your careful review of the wording in the text. Following your suggestion, "significant fluorescence" has been revised to "a prominent fluorescence signal".

**Reviewer #2:**

**Methods**

**Q1** Is the population clearly described and appropriate for the hypothesis being tested?

NO. While the authors state that the parasite was identified as *S. mansoni* using the genotyping method of Kuchta et al. 2021, they do not present any results from this analysis—such as the gene marker used, GenBank accession numbers, phylogenetic placement, or sequence similarity.

**Response:** Thank you for your comment. For the identification of plerocercoids, we used the genotyping method described in Kuchta et al. (2021). Specifically, the mitochondrial cytochrome c oxidase subunit 1 (*cox*1) gene was amplified and bidirectionally sequenced. Sequence alignment revealed that the cox1 sequence of the plerocercoid isolate used in this study was identical to the KY114886 sequence within the *Spirometra mansoni* clade in the phylogenetic analysis by Kuchta et al. (2021). Therefore, both sequence alignment and phylogenetic analysis support that the plerocercoid used in this study was *Spirometra mansoni*.

Kuchta R, Kołodziej-Sobocińska M, Brabec J, Młocicki D, Sałamatin R, Scholz T. Sparganosis (*Spirometra*) in Europe in the Molecular Era. Clin Infect Dis. 2021;72: 882-890.

**Results**

**Q2** Are the results clearly and completely presented?
NO. The main results are quite clear and follow a logical structure. However, some areas lack detail or supporting data—for example, species identification of *S. mansoni* is stated but not shown. The manuscript repeatedly refers to the use of the *S. mansoni* genome, which has not been sequenced; only in one instance is an accession number provided, which actually corresponds to *S. erinaceieuropaei*, indicating inconsistency and a need for clarification. Also DEG numbers sometimes differ between text and figures. The rationale for focusing on coexpressed DEGs is also not fully explained. The manuscript does not state where the sequencing data will be made available. To ensure transparency and reproducibility, the authors should clearly indicate the repository (e.g., NCBI SRA, ENA, or CNGBdb) where the raw transcriptomic data will be deposited, along with relevant accession numbers.

**Response:** Thank you very much for your comments. We have summarized your questions, checked and revised them one by one, and now reply as follows: 1) Regarding the identification of plerocercoids, we used the genotyping method described in Kuchta et al. (2021). Specifically, the mitochondrial cytochrome c oxidase subunit 1 (*cox*1) gene was amplified and bidirectionally sequenced. Sequence alignment revealed that the cox1 sequence of the plerocercoid isolate used in this study was identical to the KY114886 sequence within the *Spirometra mansoni* clade in the phylogenetic analysis by Kuchta et al. (2021). Therefore, both sequence alignment and phylogenetic analysis support that the plerocercoid used in this study was *Spirometra mansoni*.

2) Regarding the issue of the reference genome, although the reference genome used in this study is that of *Spirometra erinaceieuropaei* (GCA_902702965.1) (Kikuchi et al., 2021), recent taxonomic studies have revealed that most *Spirometra* isolates from Asia have been misidentified (Kuchta et al., 2021). Specifically, the Asian isolates previously identified as *S. erinaceieuropaei* should actually be classified as *S. mansoni*, whereas the true *S. erinaceieuropaei* is distributed only in certain areas of Europe. Consequently, the parasite strain employed in this study belongs to the same species as the one used for the reference genome. Nevertheless, to maintain consistency with the original literature, we continue to use the name *S. erinaceieuropaei* for the reference genome in our study.

Kikuchi T, Dayi M, Hunt VL, Ishiwata K, Toyoda A, Kounosu A, Sun S, Maeda Y, Kondo Y, de Noya BA, Noya O, Kojima S, Kuramochi T, Maruyama H. Genome of the fatal tapeworm Sparganum proliferum uncovers mechanisms for cryptic life cycle and aberrant larval proliferation. Commun Biol. 2021;4(1):649.

Kuchta R, Kołodziej-Sobocińska M, Brabec J, Młocicki D, Sałamatin R, Scholz T. Sparganosis (*Spirometra*) in Europe in the Molecular Era. Clin Infect Dis. 2021; 72: 882-890

3) To address the issue of the number of differentially expressed genes (DEGs), we have reviewed the DEG data in the text and figures and corrected the inconsistencies between the text and the figures.

4) Regarding the rationale for focusing on coexpressed differentially expressed genes (DEGs), since this study aims to reveal the core regulatory factors involved in the proglottid development of *Spirometra mansoni*, priority was given to the analysis of coexpressed DEGs, as these genes are more likely to play more critical roles in the process of proglottid differentiation. In addition, we have provided specific descriptions of the database where the original transcriptome data are stored, as well as the relevant accession numbers. The RNA-seq data from this study have been deposited in the NCBI Sequence Read Archive (SRA accession number PRJNA1248218) and are accessible through accession numbers SAMN47853541, SAMN47853542, SAMN47853543, SAMN47853544, SAMN47853545, SAMN47853546, SAMN47853547, SAMN47853548, and SAMN47853549.

**Q3** Are the figures (Tables, Images) of sufficient quality for clarity?

No. while the overall quality and resolution of figures is acceptable, Figure 2A raises concerns. Specifically, some bars representing DEGs are taller than those labeled “All,” which is conceptually inconsistent, as DEGs should form a subset of all annotated genes. This suggests possible mislabeling or unclear grouping of data. The figure would benefit from clearer explanation in the legend or main text to avoid misinterpretation.

**Response:** Thank you for your suggestion. Regarding the visual contradiction in Figure 2A, we would like to add the following explanation: the right vertical axis represents the number of genes, and each axis scale corresponds to two values, where the blue value indicates the number of DEGs and the black value indicates the number of all genes. Owing to the different scale ranges of the vertical axis, some DEG columns visually appear higher than the "All" columns, but in actual values, DEGs are still a subset of all genes.

**Q4** Figure 6A refers to “chromosomal localization” of SmPKs. However, the authors used the fragmented draft genome of *Spirometra erinaceieuropaei*, which is not at chromosome level. Therefore, this panel is misleading. The term “scaffold-level localization” would be more accurate, or clarification in the figure legend and methods is needed to avoid overstating the genome assembly quality. The figure legends in the manuscript are generally overly brief and lack sufficient detail.

**Response:** Following your recommendations, we have made the following revisions to Figure 6: 1) Terminology correction: "Chromosomal localization" in Figure 6A has been replaced with "scaffold-level localization". All relevant text has been updated by changing "chromosomes" to "scaffolds". 2) Method modification: The original description "To precisely map *Sm*PK genes on chromosomes, distribution information was collected from the *S. mansoni* genome, and gene distribution maps were generated using TBtools2 software" has been revised to "To precisely map *Sm*PK genes on scaffolds, distribution information was collected from the *S. erinaceieuropaei* draft genome (NCBI accession GCA_902702965.1), and gene distribution maps were generated using TBtools2 (v2.136) software". 3) Figure legend modification: The original legend "Figure 6A. Chromosomal localization: the green columns represent different chromosomes of *S. mansoni*" has been revised to "Scaffold-level localization: the green columns represent different scaffolds of *S. erinaceieuropaei*". The revised Figure 6 is below:

**Q5** As they currently stand, the figures cannot be fully understood without referring to the main text, which limits their standalone interpretability. This is particularly problematic for readers who rely on figure legends to quickly grasp experimental results. For example, the legend for Figure 3B simply states: “B. GSEA of the glycolysis/gluconeogenesis pathway.” This provides no information on: what comparison was made (e.g., which proglottid stages were analyzed), what the enrichment score represents, or how the result should be interpreted in a biological context. More informative and self-contained figure legends are strongly recommended.

**Response:** Thank you for your suggestion. To address the issues of overly brief figure legends and insufficient independent interpretability, we have systematically revised all the figure legends, with a focus on adding key points for result interpretation to ensure that the figures and tables can be understood independently of the main text. Specifically, for Figure 3B, the revised figure legend is as follows: "**B.** Gene set enrichment analysis of the glycolysis pathway in the SNIPs and MPs. A positive ES indicates significant enrichment of this pathway in MPs. "

**Conclusions**

**Q6** Are the limitations of analysis clearly described?

NO. The manuscript does not adequately discuss the limitations of the analysis. Important aspects such as the use of a genome from *S. erinaceieuropaei* instead of *S. mansoni*, the lack of independent validation of species identity, absence of functional knockdown experiments to confirm gene function, and potential limitations of in vitro assays are not addressed. A more transparent discussion of these issues would strengthen the credibility and interpretation of the results.

**Response:** Thank you for your valuable suggestions. First, regarding the issues of the reference genome and species identification, as mentioned earlier, the species of the reference genome we used and the parasite strain in this study are actually the same species, namely, *Spirometra mansoni*. Second, concerning the problems of gene function verification and potential limitations of in vitro experiments, following your suggestions, we have made the following revisions. Admittedly, functional knockout experiments are a classic method for verifying gene function. However, *Spirometra mansoni*, as a multicellular parasite with a complex life cycle, presents significant technical challenges for conducting gene knockout studies at the adult stage. This remains a common obstacle in current research within this field: 1) Difficulty in obtaining adult samples. Adults parasitize the intestines of definitive hosts (such as cats and dogs). Artificial infection of definitive hosts requires 3–6 months of rearing before the ingested spargana develop into adult worms. Each experiment typically yields only 2–3 adult worms, which is insufficient to meet the sample size and reproducibility requirements for functional knockout studies. 2) Lack of a long-term in vitro culture system. Functional studies of parasites rely on stable in vitro culture systems. However, adult worms are highly sensitive to environmental conditions and require strict simulation of the intestinal microenvironment of definitive hosts (such as specific pH values, anaerobic conditions, and host secretory factors). To date, no internationally established long-term in vitro culture method supports the survival of adult worms, making it impossible to observe postknockout phenotypes over extended periods. Despite the absence of adult-stage knockout experiments, this study preliminarily verified the function of pyruvate kinase (PK) through in vitro enzyme inhibition assays. However, potential limitations include the following: 1. Sample constraints prevented the testing of multiple inhibitor concentration gradients. 2. Interindividual variability may exist between the experimental and control groups.

**Q7** Is public health relevance addressed?

NO. Although *Spirometra mansoni* is a zoonotic parasite with public health importance, the manuscript does not discuss the relevance of the findings in the context of human infection or disease.

**Response:** Thank you for your comment. We have revised the text as follows: “This study provides the first comprehensive characterization of gene expression profiles across different proglottids of *S. mansoni.* PK plays a pivotal role in proglottid differentiation, laying the foundation for further exploration of the differentiation mechanism of segments in tapeworms.”

**Editorial and Data Presentation Modifications**

**Abstract:**

**Q8** Please specify which types of proglottids were analyzed.

**Response:** Thank you for your comment. The types of proglottids have been revised as “...3 types of proglottids (scolex-neck-immature proglottids, SNIPs; mature proglottids, MPs; and gravid proglottids, GPs)...” in the revised MS.

**Q9** Several sentences in the abstract and author summary are identical. This redundancy should be eliminated. Redundant example: "Phylogenetic analysis revealed that SmPKs have undergone varying degrees of evolution and exhibit high diversity. The optimal reaction conditions for recombinant SmPK1 (rSmPK1) were 37°C and pH 8.0, and the addition of K+/Mg2+ significantly enhanced its catalytic activity. Tannic acid could significantly inhibit the activity of SmPK1 in vitro..." This is repeated verbatim in both sections with only minor terminological changes. Please revise to avoid duplication.

**Response**: Thank you for your comment. Following your suggestion, we have revised this part of the content as follows: Phylogenetic analysis revealed that *Sm*PKs exhibit high diversity and are scattered across different cestode groups. Enzyme activity assays confirmed that recombinant *Sm*PK1 exhibits native enzymatic activity and that tannic acid exerts specific, dose-dependent inhibitory effects on r*Sm*PK1. Further in vitro inhibition experiments using tannic acid revealed that pyruvate kinase activity was continuously inhibited, accompanied by a consistent and synchronous decrease in pyruvate content, a time-dependent consumption of triglycerides, and a gradual depletion of total sugar reserves. These results suggest that pyruvate kinase may influence tapeworm proglottid differentiation by regulating the glycolytic pathway.

**Introduction:**

**Q10** The introduction should mention pyruvate kinase (PK) and its importance in parasite metabolism, given its central role in the study.

**Response:** Thank you for your comment. This content has been supplemented in the introduction according to your suggestion. Among the genes related to energy metabolism, pyruvate kinase (PK) is a key regulatory factor that controls metabolic flux and ATP production during glycolysis in parasites. Glycolysis is the core pathway by which cells utilize glucose to produce energy, which serves as the primary energy source for parasites (Bonive-Boscan et al., 2024). In this process, glucose is converted to pyruvate (PYR) via an oxygen-independent pathway, with the generation of ATP. The multifunctional metabolite PYR participates in subsequent aerobic and anaerobic oxidation, providing the essential energy for cellular activities (Gray et al., 2015). The rate of glucose conversion to PYR is coregulated by pyruvate kinase (Schormann et al., 2019). In the final step of glycolysis, PK irreversibly catalyses the transfer of phosphate from phosphoenolpyruvate (PEP) to adenosine diphosphate (ADP), generating pyruvate and ATP and regulating cellular energy production (Ayyildiz et al., 2020). To date, PK has been isolated from various parasites, and it plays important roles in parasite‒host interactions, parasite survival, and parasite development (Khan et al., 2022; Yue et al., 2022).

Bonive-Boscan A.D., Acosta H., Rojas A., Metabolic changes that allow *Plasmodium falciparum* artemisinin-resistant parasites to tolerate oxidative stress, Front Parasitol. (2024) 3:1461641.

Gray LR, Sultana MR, Rauckhorst AJ, Oonthonpan L, TomPyKins SC, Sharma A, et al. Hepatic mitochondrial pyruvate carrier 1 is required for efficient regulation of gluconeogenesis and whole-body glucose homeostasis. Cell Metab. 2015; 22(4): 669–81. pmid:26344103

Schormann N, Hayden KL, Lee P, et al. An overview of structure, function, and regulation of pyruvate kinases [J]. Protein Sci, 2019, 28(10): 1771-84.

Ayyildiz M, Celiker S, Ozhelvaci F, Akten ED. Identification of Alternative Allosteric Sites in Glycolytic Enzymes for Potential Use as Species-Specific Drug Targets. Front Mol Biosci. 2020;7:88.

Khan SM, Zhang X, Witola WH. *Cryptosporidium parvum* Pyruvate Kinase Inhibitors With in vivo Anti-cryptosporidial Efficacy. Front Microbiol. 2022;12:800293.

Yue WW, Yan SW, Zhang R, Cheng YK, Liu RD, Long SR, Zhang X, Wang ZQ, Cui J. Characterization of a novel pyruvate kinase from *Trichinella spiralis* and its participation in sugar metabolism, larval molting and development. PLoS Negl Trop Dis. 2022;16(10):e0010881.

**Q11** Latin binomials (e.g., *Spirometra mansoni*) should be spelled in full on first mention (lines 95-96).

**Response:** We have made this revision in accordance with your suggestions.

**Q12** The section on nanopore sequencing (lines 102–114) is tangential and could be condensed or removed.

**Response:** Thank you for your suggestions. We have streamlined this part of the content: “compared with traditional short-read sequencing technologies, nanopore sequencing for full-length transcriptome characterization offers significant advantages: (1) Nanopore sequencing can avoid the loss of transcript information from fragmentation, thereby increasing the accuracy of quantification [10]; (2) it enables structural characterization of transcriptional events, facilitating comprehensive molecular mechanism analysis [11]; and (3) it requires minimal sample input, making it particularly advantageous for studies with limited sample availability [12]. In recent years, an increasing number of studies have utilized nanopore sequencing for whole-genome or transcriptome analyses of parasites, such as *Caenorhabditis elegans*, *Clonorchis sinensis*, *Trypanosoma cruzi*, and *Plasmodium falciparum*[13-16].”

**Q13** The sentence identifying the proglottid types (lines 116-117) should be incorporated into the abstract.

**Response:** Thank you for your suggestion. We have made revisions in accordance with your advice.

**Parasites and Animals:**

**Q14** The species identification via Kuchta et al.'s method is stated but no supporting data are provided. Include: Gene marker used. GenBank accession numbers. Sequence similarity or phylogenetic placement. At minimum, include this data in a supplementary table.

**Response:** Thank you for your suggestion. As mentioned above, we identified the parasites used in the present study using the *cox*1 gene. Following your advice, we have supplemented the sequencing results in Supplementary Table S1, and the specific sequence information is as follows:

>Seq_*cox*1

ATGGCTAAGTTTAGTTTTTTTAGTTGGCTTTTTACGCTAGATCATAAGCGTGTGGGTATGATTTATACTTTAATTGGGATATGATCAGGTTTTGTTGGGTTGAGTTTTAGGGTAATGATACGTGTTAATTTCGTTGAGCCTTATTTTAATGTAATTTCTTCGGACTGTTATAAATTTTTGATAACTAATCATGGTATTATTATGATTTTTTTTTTTTTGATGCCTGTGTTGATTGGGGGTTTTGGTAATTATTTGATTCCTTTATTATCCGGTTTACCAGATTTAAATTTACCTCGGTTGAAAGCCTTGAGAGCATGGTTGCTTTTTCCTTCTATTTTATTTCTTGTGCTGAGTATGTGTTTGGGTGCTGGTATAGGGTGGACTTTTTATCCCCCTCTCTCCTCTTCTCTTTTTAGGGATAGTCGTGGTGTTGATCTTTTGATGTTTTCTTTACATTTGGCTGGTCTTTCTAGTTTGTTGGGTTCTATAAATTTTATATGTACTCTTTACTCAGCTTTTGTTGATAATTTTGTGTCTCGTAGTTCTATTTTGTTGTGGTCTTATCTATTTACTTCTATTCTTTTATTGTTAACTATTCCTGTGTTGGCTGCTGCCATTACAATGTTGTTGTTTGATCGTAAATTTGGTTCGGCTTTTTTTGATCCTTTGGGTGGTGGTGATCCTGTTTTGTTTCAACATATGTTTTGGTTTTTTGGGCATCCAGAAGTTTATGTATTGATTTTGCCTGGTTTTGGTATGGTAAGTCATGTGTGTAGTAAATTAGGTTGTTCATATGATACTTTTGGTTTTTATGGTTTATTATTTGCTATGTTTTCTATAGTGTGTTTAGGTAGCGTGGTTTGGGGTCACCACATGTTTACTGTGGGGTTGGACGTGAAGACTGCTGTTTTCTTTAGTTCTGTGACTATGATTATTGGGGTTCCCACGGGTATAAAGGTGTTTTCTTGGCTTTATATGATTTTAAATAGTCGTGTTTCGTTGCGTGAGCCTGTGTTTTGATGGGTTTTATCCTTTATTGTGTTGTTTACTATGGGTGGTGTTACTGGTATAATTCTTTCTGCTTGTGTGTTGGATAAAATTTTGCATGACACGTGGTTTGTGGTGGCTCATTTTCATTATGTTATGTCTTTGGGTTCTTATATTAGGGTTATTATATTTTTTGTTTGGTGATGGCCTGTTATCACAGGGGTTAGCTTGAATAAGTATTTGTTACAGTGTCATTGTATAGTATCAAATGTGGGCTTTAATTTGTGTTTTTTTCCTATGCATTATTTTGGTATTTGTGGTTTACCTCGGCGTGTTTGTGTGTATGAGTCAGGGTACGCTTGAGTTAATATGCTTTGTTCAATAGGTTCTTTTGTTTCTGCCTTTAGTGGTTGCTTTTTTATTTTTATTTTATGGGAGTCTTTAGCTAAAAAGAATGTTGTTATAGGTTATTATGGTAGTTCTTCAACTTTGCTTAATTTGTGTTGATCGCCAGTGCCTTACCACAGTAATTTTTTTGTGCGTGGATTATTTGTTGATTATTCTGTATTGGCTTTTTAG

**Materials and Methods:**

**Q15** Line 145: CoWin Biosciences is a distributor; the TRIzol manufacturer is Invitrogen.

**Response:** According to your suggestion, the TRIzol manufacturer has been revised to “Invitrogen”.

**Q16** Lines 152–155: Missing critical information about software tools for quality filtering, rRNA removal, primer detection, and FLNC identification. Please add.

**Response:** Thank you for your comments. We have added the relevant content. The quality filtering tool used was MinKNOW version 2.2 (Oxford, United Kingdom), the ribosomal RNA filtering tool used was Minimap2 + SAMtools, and the tool for primer detection and FLNC identification was Pychopper (v.2.4.0).

**Q17** Lines 161–165: Clarify how CPM was used. DESeq2 does not use CPM for normalization.

**Response:** On the basis of your suggestions, the relevant section has been revised to: Mapped full-length reads with > 5 match quality were chosen for quantification. To ensure that read counts accurately reflect gene expression levels, CPM (counts per million) was employed as the metric for quantifying gene expression [27]. Differential expression analysis among different proglottids was conducted with the DESeq R package (1.6.3), which is based on a negative binomial distribution model to analyse raw count data. The false discovery rate (FDR) was adjusted and controlled with the method of Benjamini and Hochberg, and DEGs with a log2fold change (FC) ≥ 2 and an FDR < 0.01 were chosen.

**Q18** Lines 168–176 (Annotation and Enrichment): Specify database versions or download dates. Justify use of both COG and KOG. Name the GSEA software/tool used. Include STRING database version and confidence threshold. Provide Cytoscape version.

**Response:** Thank you for your suggestion. We have added the following relevant content: Databases such as the NR (http://www.ncbi.nlm.nih.gov/), SwissProt (http://www.uniprot.org/), GO (http://www.geneontology.org/), COG (http://www.ncbi.nlm.nih.gov/COG/), KOG (http://www.ncbi.nlm.nih.gov/KOG/), Pfam (http://pfam.xfam.org/), eggNOG (http://eggnogdb.embl.de) and KEGG (http://www.genome.jp/kegg/) databases were used for gene function annotation. Gene ontology enrichment analysis of DEGs was conducted using the GOseq R package-based Wallenius noncentral hypergeometric distribution [28]. KEGG pathway enrichment analysis of DEGs was performed with KEGG Orthology-Based Annotation System (KOBAS) software [29]. Gene set enrichment analysis (GSEA) was conducted using R/clusterProfiler (v4.4.4) to identify gene sets associated with cestode development, with thresholds set as NES > 1, NOM *p* value < 0.01, and FDR *q* value < 0.25. Protein‒protein interactions (PPIs) for all detected DEGs were predicted using the STRING database (v10.5) with a default threshold of 0.4 and visualized via Cytoscape (v3.10.0). 3) *Spirometra mansoni* is a eukaryote. KOG, designed for eukaryotes, enables inference of the worm's gene functions through its classification of eukaryotic orthologous groups. Although COG is prokaryote-based, it contains partial eukaryotic data, which can assist in cross-species functional comparisons. The combination of both is consistent with its eukaryotic properties and functional annotation needs.

**Q19** Lines 194–202: The discussion of PK function belongs in the Introduction. The Methods should focus on identification criteria for SmPKs.

**Response:** Thank you for your suggestion. This content has been supplemented in the introduction per your recommendation. Among the genes related to energy metabolism, pyruvate kinase (PK) is a key regulatory factor that controls metabolic flux and ATP production during glycolysis in parasites. Glycolysis is the core pathway by which cells utilize glucose to produce energy and serve as the primary energy source for parasites (Bonive-Boscan et al., 2024). In this process, glucose is converted to pyruvate (PYR) via an oxygen-independent pathway, with the generation of ATP. The multifunctional metabolite PYR participates in subsequent aerobic and anaerobic oxidation, providing the essential energy for cellular activities (Gray et al., 2015). The rate of glucose conversion to PYR is coregulated by pyruvate kinase (Schormann et al., 2019). In the final step of glycolysis, PK irreversibly catalyses the transfer of phosphate from phosphoenolpyruvate (PEP) to adenosine diphosphate (ADP), generating pyruvate and ATP and regulating cellular energy production (Ayyildiz et al., 2020). To date, PK has been isolated from various parasites, and it plays important roles in parasite‒host interactions, parasite survival, and parasite development (Khan et al., 2022; Yue et al., 2022).

Bonive-Boscan A.D., Acosta H., Rojas A., Metabolic changes that allow *Plasmodium falciparum* artemisinin-resistant parasites to tolerate oxidative stress, Front Parasitol. (2024) 3:1461641.

Gray LR, Sultana MR, Rauckhorst AJ, Oonthonpan L, TomPyKins SC, Sharma A, et al. Hepatic mitochondrial pyruvate carrier 1 is required for efficient regulation of gluconeogenesis and whole-body glucose homeostasis. Cell Metab. 2015; 22(4): 669–81. pmid:26344103

Schormann N, Hayden KL, Lee P, et al. An overview of structure, function, and regulation of pyruvate kinases [J]. Protein Sci, 2019, 28(10): 1771-84.

Ayyildiz M, Celiker S, Ozhelvaci F, Akten ED. Identification of Alternative Allosteric Sites in Glycolytic Enzymes for Potential Use as Species-Specific Drug Targets. Front Mol Biosci. 2020;7:88.

Khan SM, Zhang X, Witola WH. *Cryptosporidium parvum* Pyruvate Kinase Inhibitors With in vivo Anti-cryptosporidial Efficacy. Front Microbiol. 2022;12:800293.

Yue WW, Yan SW, Zhang R, Cheng YK, Liu RD, Long SR, Zhang X, Wang ZQ, Cui J. Characterization of a novel pyruvate kinase from *Trichinella spiralis* and its participation in sugar metabolism, larval moulting and development. PLoS Negl Trop Dis. 2022;16(10):e0010881.

**Q20** Lines 211–213: Clarify genome source. Mapping was done against *S. erinaceieuropaei* (line 157), not *S. mansoni*. Unify terminology and remove misleading statements.

**Response:** Thank you for your suggestion. This section has been revised to “To precisely map *Sm*PK genes on scaffolds, distribution information was collected from the *S. erinaceieuropaei* draft genome (NCBI accession GCA_902702965.1), and gene distribution maps were generated using TBtools2 (v2.136) software”.

**Q21** Lines 213–216: Provide software version for TBtools2.

**Response:** The software version for TBtools2 has been added: TBtools2 (v2.136).

**Q22** qRT-PCR Section: Include summarized protocol (referencing Liu et al. is fine). Clarify whether GAPDH stability was verified. State whether technical replicates were used.

**Response:** Thank you for your comment. GAPDH is a housekeeping gene in metazoans. We chose GAPDH as a reference gene because it is stably expressed and has been used as a reference gene in many studies (Kondo et al., 2020; Chen et al., 2022). In addition, the expression of GAPDH is stable at different developmental stages of *S. mansoni*. For the qRT‒PCR analysis, three biological replicates were used, and differences were considered statistically significant at *p* < 0.05.

Kondo Y, Ito D, Tademoto S, Itami N, Nishikata S, Takashima E, Tsuboi T, Fukumoto S, Otsuki H. Molecular cloning and characterization of plerocercoid-immunosuppressive factor from *Spirometra erinaceieuropaei*. Parasitol Int. 2020;76:102062.

Chen WQ, Liu SS, Cheng C, Cui J, Wang ZQ, Zhang X. Molecular characteristics of glutathione transferase gene family in a neglect medical *Spirometra* tapeworm. Front Vet Sci. 2022;9:1035767.

**Q23** Line 226: Use "medically important cestodes" instead of "medical cestodes".

**Response:** The phrase has been revised according to your suggestion.

**Q24** Line 285: The gene count (26,270) seems inflated. Clarify if this includes isoforms, all transcripts, or coding genes. Define criteria for gene prediction.

**Response:** Thank you for your question. Following your suggestion, we have conducted a further check and confirmed that the gene count is 16501, including all expressed transcripts.

**Q25** Line 286: Define "novel genes" and the method of identification (e.g., BLAST, absence in reference genome).

**Response:** Thank you for your question. In the analysis, we obtained a gff3 file after removing redundancy from the full-length transcripts. We then used gffcompare software to compare this file with the gff3 file of the reference genome and selected the transcripts and genes that were identified in this sequencing but not annotated in the reference genome as novel transcripts and novel genes.

**Q26** GO and KEGG Analysis: Focus only on co-expressed DEGs may overlook stage-specific signals. Include condition-specific analyses.

**Response:** Thank you for your important suggestions. Since this study aims to reveal the core regulatory factors involved in the development of *Spirometra mansoni*, priority was given to the analysis of coexpressed DEGs, as these genes are more likely to play more critical roles in the process of proglottid differentiation.

**Q27** Figure 2A: The DEGs bar height exceeding the "All" bar is misleading. Clarify calculation or replot with enrichment (e.g., fold change or p value).

**Response:** Thank you for your suggestion. Regarding the visual contradiction in Figure 2A, we would like to add the following explanation: the right vertical axis represents the number of genes, and each axis scale corresponds to two values, where the blue value indicates the number of DEGs and the black value indicates the number of all genes. Owing to the different scale ranges of the vertical axis, some DEG columns visually appear higher than the "All" columns, but in actual values, DEGs are still a subset of all genes.

**Q28** Venn Diagram vs DEG Counts: Numbers in Venn diagrams (Fig. S1a) differ from those in text. If only annotated genes are shown, clarify this.

**Response:** Thank you for pointing out this issue. We have revised this part of the content and provided relevant tables in the supplementary materials (Supplementary Tables 5–8).

**Q29** Same applies for KEGG: counts in text differ from Fig. 2a. Clarify filtering criteria and ensure consistency.

**Response:** Thank you for your suggestion. Regarding the visual contradiction in Figure 2A, we would like to add the following explanation: the right vertical axis represents the number of genes, and each axis scale corresponds to two values, where the blue value indicates the number of DEGs and the black value indicates the number of all genes. Owing to the different scale ranges of the vertical axis, some DEG columns visually appear higher than the "All" columns, but in actual values, DEGs are still a subset of all genes. In addition, only the top 50 KEGG pathways with the largest number of genes are shown in Fig. 2C.

**Q30** Species Identification: Despite claims, the S. mansoni genome does not exist. The provided accession number matches *S. erinaceieuropaei*. This must be corrected throughout.

**Response:** Thank you very much for your comments. Although the reference genome used in this study was *Spirometra erinaceieuropaei* (GCA_902702965.1) (Kikuchi et al., 2021), recent taxonomic studies (Kuchta et al., 2021) revealed that most *Spirometra* isolates from Asia were misidentified. Specifically, the Asian isolates previously identified as *S. erinaceieuropaei* should actually be classified as *S. mansoni*, while the true *S. erinaceieuropaei* is distributed in only certain parts of Europe. Consequently, the parasite strain employed in this study belongs to the same species as the one used for the reference genome. Nevertheless, to maintain consistency with the original literature, we continue to use the name *S. erinaceieuropaei* for the reference genome in this research.

Kikuchi T, Dayi M, Hunt VL, Ishiwata K, Toyoda A, Kounosu A, Sun S, Maeda Y, Kondo Y, de Noya BA, Noya O, Kojima S, Kuramochi T, Maruyama H. Genome of the fatal tapeworm Sparganum proliferum uncovers mechanisms for cryptic life cycle and aberrant larval proliferation. Commun Biol. 2021;4(1):649.

Kuchta R, Kołodziej-Sobocińska M, Brabec J, Młocicki D, Sałamatin R, Scholz T. Sparganosis (*Spirometra*) in Europe in the Molecular Era. Clin Infect Dis. 2021; 72: 882-890

**Summary and General Comments**

**Q31** This manuscript explores the role of pyruvate kinase in the segmental differentiation of *Spirometra mansoni* through full-length transcriptomic profiling and complementary biochemical analyses. The topic is timely and relevant, especially given the limited understanding of energy metabolism in cestode development. The use of Nanopore sequencing alongside functional assays provides a potentially valuable dataset and offers insights into metabolic regulation during proglottid maturation. However, the overall execution of the study falls short in several key areas. Methodological descriptions are often lacking in detail, especially concerning the source of genomic data, parasite species confirmation, and analysis reproducibility.

**Response:** Thank you very much for your comments. Following your suggestions, in the revised manuscript, we have carefully checked and revised issues such as the data source of the genome, the identification of parasite species, and the reproducibility of the analysis.

**Q32** Critical background information (such as the identity and validation of the species used) is not adequately documented, which undermines confidence in the conclusions.

**Response:** Thank you for your suggestions. This part of the content has been supplemented and revised.

**Q33** Moreover, the interpretation of data sometimes overreaches the presented evidence, with speculative links made between metabolic changes and differentiation in the absence of causal functional validation.

**Response:** Thank you for your comments. Following your suggestions, we have revised this part of the content as follows: “In this process, PK1 acts as a core regulator of metabolic reprogramming, playing a critical role in modulating the energy metabolism of the cestode. Interestingly, transcriptomic analysis revealed the progressive upregulation of *Sm*PK1 expression from SNIPs to MPs and GPs, paralleling the increased activity of the glycolysis pathway. Given the pivotal role of PK in glucose metabolism, we propose that *Sm*PK1 may regulate cestode segment differentiation and maturation by modulating the glycolysis pathway.”

**Q34** The manuscript would also benefit from clearer and more informative figure legends, consistent use of terminology, and removal of duplicated or overly general text across sections. Some figures are misleading in how data are labeled or described, and others lack standalone clarity. In particular, the reliance on a genome from *S. erinaceieuropaei*—without consistent disclosure or justification—requires correction and clarification.

**Response:** We sincerely appreciate your valuable comments. Following your suggestions, we have reviewed, supplemented, and revised all the figure captions.

**Reviewer #3:**

**Results**

**Q1** it would be helpful to provide results such as PCA plots, hierarchical clustering heatmaps, or pairwise correlation analyses to demonstrate the consistency and expected separation among the biological replicates. Including this information would improve the transparency of the data quality control and can help increase the overall reliability of the differential expression results.

**Response:** Thank you for your valuable suggestions. In fact, principal component analysis (PCA) was performed during our data analysis process, although it was not included in the original manuscript. In response to your recommendation, we have supplemented this analysis with an appendix in the revised manuscript. The PCA results demonstrated that biological replicates within the same group clustered tightly together, while distinct separation was observed between different groups, with no abnormal outliers detected.

**Q2** I would also encourage the authors to check whether their Nanopore long-read data enabled the identification of any novel isoforms or previously unannotated gene structures. Presenting such findings, even briefly as supplementary information, would further demonstrate the added value of using Nanopore full-length sequencing and would make the dataset more useful for future studies on *S. mansoni* transcriptome complexity.

**Response:** Thank you for your suggestions. We have revised this part of the content: “compared with traditional short-read sequencing technologies, nanopore sequencing for full-length transcriptome characterization offers significant advantages: (1) Nanopore sequencing can avoid the loss of transcript information from fragmentation, thereby increasing the accuracy of quantification [10]; (2) it enables structural characterization of transcriptional events, facilitating comprehensive molecular mechanism analysis [11]; and (3) it requires minimal sample input, making it particularly advantageous for studies with limited sample availability [12]. In recent years, an increasing number of studies have utilized nanopore sequencing for whole-genome or transcriptome analyses of parasites, such as *Caenorhabditis elegans*, *Clonorchis sinensis*, *Trypanosoma cruzi*, and *Plasmodium falciparum*[13-16].”

**Q3** For Figure 8B, please include statistical significance testing for the qPCR results to confirm that the observed differences are robust.

**Response:** Thank you for your suggestion. In this figure, we focused primarily on illustrating the expression patterns of *Sm*PK1 across different life cycle stages of *Spirometra mansoni* and therefore did not perform statistical significance testing.

**Conclusions**

**Q4** The study demonstrates, through comparative transcriptomic analysis, enzyme activity assays, and inhibitor experiments, that PKs play a key role in the energy metabolism of tapeworms. However, it would be helpful for the authors to explicitly acknowledge that direct experimental evidence linking PKs to the regulation of differentiation through glycolysis is still lacking and that this remains a potential area for future study. I therefore recommend that the authors refine the title to more precisely reflect the scope of the study’s findings.

**Response:** Thank you for your suggestions. We fully agree with your views, and the title has now been revised to "Transcriptomic analysis reveals that pyruvate kinase potentially plays a key role in the differentiation of *Spirometra mansoni* proglottids by regulating the glycolysis pathway".

**Editorial and Data Presentation Modifications**

**Q5** the temperature unit in line 71 is formatted correctly by adding a space between the numeric value and the degree Celsius symbol (e.g., “37 °C” instead of “37°C”). In addition, the same issue exits in the Method section.

**Response:** Thank you for your suggestions. We have made the changes you requested and have reviewed and revised the entire manuscript.

**Q6** I suggest that the authors expand the Introduction to include a brief overview of relevant previous studies that have used omics approaches to investigate the molecular mechanisms of differentiation in sparganosis. This additional context would help highlight the knowledge gap addressed by the present study and emphasize the novelty and significance of applying full-length transcriptomic analysis to *S. mansoni*.

**Response:** Thank you for your suggestions. We have revised the manuscript in accordance with your recommendations.

**Summary and General Comments**

**Q7** I recommend that the authors provide a brief description in the Introduction section explaining the biological differences among the three segments used for sequencing (scolex-neck-immature proglottid, mature proglottid, and gravid proglottid). This background would help readers better understand the biological context and developmental progression of *Spirometra mansoni* proglottids and the rationale for selecting these segments for comparative transcriptomic analysis. Adding this context will help readers unfamiliar with tapeworm biology better understand the link between segmental morphology and the regulation of differentiation.

**Response:** Thank you very much for your affirmation of this study. Regarding the issue of "in-depth interpretation of tapeworm physiology-related characteristics", we have added the following relevant content in the revised manuscript: "Adult *S. mansoni* show characteristic body differentiation into the scolex, neck and strobila. The neck section is a very small part that occurs after the scolex section, and its function is to continuously bud off the segments. The strobila segments can be divided into immature proglottids (IPs), mature proglottids (MPs) and gravid proglottids (GPs) according to their anteroposterior position and the development of their sexual organs. Just behind the neck are the IPs, and further from the posterior are MPs with mature female and male organs and, finally, those whose uterus contains fertilized eggs. The latter proglottids are described as GPs. The gravid or senile terminal proglottids detach or disintegrate [5, 6]."

Conboy G. Cestodes of dogs and cats in North America. Vet Clin North Am Small Anim Pract. 2009;39(6):1075-90, vi.

Bo X, Zhao W, Zhang H, Kang L, Wang X. Gene expression profiling of in *Moniezia expansa* at different developmental proglottids using cDNA microarray. Mol Biol Rep. 2012;39(4):4499-507.

**Q8** While the Introduction clearly highlights the general advantages of Nanopore long-read sequencing, I recommend that the authors briefly acknowledge that Illumina short-read sequencing generally provides more robust and established quantification for gene-level differential expression analysis in Discussion. Additionally, since the authors have already generated Nanopore data, they could consider better leveraging the unique strengths of the long-read approach by including an isoform-level analysis or using the full-length reads to refine the gene models and improve the genome annotation. Adding such information, even at a summary level, would enhance the impact of the dataset and showcase the full advantage of combining expression quantification with structural transcriptome insights.

**Response:** We sincerely appreciate your valuable suggestions. In the revised manuscript, we have incorporated the statement that "Nanopore long-read sequencing generally provides more robust and established quantification for gene-level differential expression analysis" into the Discussion section as follows: “All nine samples in this study presented relatively high-quality sequencing data (mean Q score = Q13, mapping rate > 96.95%), and the qPCR validation results were moderately correlated with the Nanopore sequencing results (*R*^2^ = 0.6471), which is consistent with the results of Huang et al. (2022) [39]. Collectively, these results indicate that nanopore long-read sequencing generally provides more robust and established quantification for gene-level differential expression analysis."

Huang M, Jiang Y, Qin R, Jiang D, Chang D, Tian Z, Li C, Wang C. Full-Length Transcriptional Analysis of the Same Soybean Genotype With Compatible and Incompatible Reactions to Heterodera glycines Reveals Nematode Infection Activating Plant Defense Response. Front Plant Sci. 2022 May 18;13:866322.

We are very grateful for your useful comments and suggestions on the language and structure of our manuscript. We have improved and made corrections in the revised manuscript. We earnestly appreciate the Editor’s/Reviewers’ warm remarks and hope that the revised manuscript will meet the requirements of this journal. Once again, thank you very much for your comments and suggestions.
