# Supplementary material for: Transcriptomic analysis reveals that pyruvate kinase potentially plays a key role in the differentiation of Spirometra mansoni proglottids by regulating the glycolysis pathway
Source: PLoS Negl Trop Dis. 2025 Oct 9;19(10):e0013570. doi: 10.1371/journal.pntd.0013570 (PMC12510601; doi:10.1371/journal.pntd.0013570)
Supplement: Attachment — Submitted filename: Response_to_comments_auresp_2.docx [file pntd.0013570.s027.docx]

**Dear Editors:**

Thank you for your letter and the reviewers’ comments on our manuscript entitled “**Transcriptomic analysis reveals that pyruvate kinase potentially plays a key role in the differentiation of *Spirometra mansoni* proglottids by regulating the glycolysis pathway (PNTD-D-25-00822R1)**”. These comments are valuable and very helpful for revising and improving our paper. We have studied the comments carefully and have revised our manuscript. We hope that the revised MS will meet your requirements. The main revisions are included in the manuscript, and the responses to the reviewers’ comments are detailed below.

**Responses to the editor’s comments**

**Q1.** Reviewer 1 has some additional suggestions for the manuscript which require an additional round of revision. Please address these carefully, and including additional literature support as described, and providing some additional justifications of inhibitor selections and concentrations, etc. For the in vitro data, the methods describe two replicates, so please be sure to mention the replicate counts and the specific statistical test used in the Fig 10 caption, and add error bars indicating the variability.

**Response:** Thank you very much for your suggestions. In the revised manuscript, we have carefully reviewed and addressed the comments from Reviewer 1. The specific modifications are as follows: We have improved the justification for the selection of inhibitors and the concentration settings and supplemented the relevant supporting literature. With respect to biological replicates, obtaining adult tapeworms is extremely challenging, as it requires the infection of a definitive host. Under laboratory conditions, the entire process demands stringent ethical approval. During our application for ethical clearance, after repeated communications, our university ethics committee permitted us to infect only one cat. Considering that a high infection dose could lead to intestinal obstruction, we ultimately infected the cat with 3 plerocercoids from the same host on the basis of previous studies. One worm was used for experiments such as qPCR and IFA, one was used for in vitro inhibition experiments, and the remaining worm served as a control. In the absence of independent replicates, these results can be considered only preliminary. Therefore, we have also addressed the limitations of the study in the Discussion section: "Notably, this study preliminarily validated the functional role of pyruvate kinase in the growth and development of *Spirometra mansoni* through in vitro enzyme inhibition experiments. However, owing to the challenges in sample availability and the lack of a long‒term in vitro culture system for the parasite, this study did not employ more advanced genetic techniques (such as RNA interference or CRISPR/Cas9 gene editing) to perform loss‒of‒function experiments for direct validation of gene function. Once a stable culture system is established in the future, further targeted genetic experiments will be conducted to provide more critical evidence for target validation." Finally, regarding the statistical testing methods, we supplemented the number of technical replicates, the statistical test method used, and the nature of the error bars in the figure legends: "Statistical analysis was performed using SPSS 26 software. Statistical significance was determined by one-way analysis of variance (ANOVA), with data derived from three technical replicates. A **p*-value < 0.05 was considered to indicate statistical significance. the error bars represent the means ± standard deviations. "

**Reviewer #1:**

**Overview**

**Q1** The work presented here by Zhou and collaborators shows a study of the differentiation of proglottids in the cestode Spirometra mansoni by transcriptomics. While the study is improved from the last version they presented, it still lacks a deep biological interpretation of features relevant to cestode biology. Below, I provide my critical assessment of the manuscript.

**Response:** Thank you very much for your positive feedback on our revised manuscript. With your help, our paper has been significantly improved. In response to the questions you raised, we have carefully and thoroughly addressed each point according to your requirements. We hope that the current revised version meets your expectations.

**Major points**

**Q2** The authors focus on the metabolism of cestodes. However, they do not mention that cestodes possess a unique metabolic system. In particular, the malate dismutation pathway is not addressed in the results. Consequently, this significant aspect of cestode metabolism is not analyzed in the work.

**Response:** Thank you very much for your comments. In accordance with your suggestions, we have included a dedicated discussion on the metabolic system of tapeworms in the revised manuscript, as detailed below: " Moreover, previous studies have confirmed that during the intestinal parasitic stage, tapeworms primarily rely on glycolysis-based fermentative metabolism. This process generates end products such as acetate and propionate, enabling highly efficient energy production at approximately 5 ATP/glucose. Functionally, this metabolic mode resembles the malate dismutation pathway, representing an evolved anaerobic energy‒generating strategy that allows tapeworms to adapt to hypoxic environments [49, 50]. Although pyruvate kinase does not directly participate in this pathway, the pyruvate it produces during glycolysis serves as a fundamental substrate for the malate dismutation pathway. This study revealed that alterations in pyruvate kinase activity directly affect the production of pyruvate in glycolysis, thereby indirectly influencing the progression of the malate dismutation pathway. Consequently, these changes impact both the energy metabolism and survival of the tapeworm."

Tielens AG. Energy generation in parasitic helminths. Parasitol Today. 1994 Sep;10(9):346-52.

Tielens AG, van Grinsven KW, Henze K, van Hellemond JJ, Martin W. Acetate formation in the energy metabolism of parasitic helminths and protists. Int J Parasitol. 2010 Mar 15;40(4):387-97.

**Q3** Additionally, there are few comparisons of the results with other studies that have applied transcriptomics to adult cestodes. Notably, it is surprising that the authors do not compare their findings with RNA-seq results from different regions of adult *Hymenolepis microstoma* (Olson et al., 2018, <https://doi.org/10.1186/s13227-018-0110-5).>

**Response:** Thank you for your valuable suggestions. In accordance with your advice, we carefully reviewed the study by Olson et al. and have supplemented the Discussion section of our manuscript with a statement regarding the consistency between the transcriptomic expression patterns observed in our study and those reported by Olson et al.: " This result aligns with the findings of a study on *Moniezia expansa* [8] and *Hymenolepis microstoma* [40], suggesting that MPs and GPs adopt highly similar metabolic and developmental strategies during the segment development of cestodes." It is important to note, however, that the two studies differ in their core objectives and analytical methodologies. Olson et al. primarily employed Gene Ontology (GO) annotation and enrichment analysis to identify core genes of interest (GOI) and used whole-mount in situ hybridization (WMISH) to validate the spatial expression patterns of these GOIs, thereby linking gene expression to tissue/organ functions. In contrast, our study first applied bioinformatics analysis to identify genes closely associated with differentiation in the tapeworm proglottid, followed by characterization and functional analysis of these core genes. More specifically, GO analysis revealed that the differentially expressed genes (DEGs) were predominantly enriched in metabolism-related terms. Subsequent KEGG enrichment analysis and gene set enrichment analysis (GSEA) further demonstrated that glycolytic pathway activity increased progressively as the proglottids developed and matured. In light of previous findings from our research group indicating highly active metabolic processes in adult tapeworms, we focused protein‒protein interaction (PPI) analysis on metabolically relevant DEGs. This approach identified pyruvate kinase (PK) as a central node within the energy metabolism-related gene network. Owing to these fundamental differences in research design and analytical emphasis, the specific results of the two studies cannot be directly compared in detail.

Olson PD, Zarowiecki M, James K, Baillie A, Bartl G, Burchell P, Chellappoo A, Jarero F, Tan LY, Holroyd N, Berriman M. Genome-wide transcriptome profiling and spatial expression analyses identify signals and switches of development in tapeworms. Evodevo. 2018 Nov 9;9:21.

**Q4** The use of tannic acid as an inhibitor for the enzyme activity assay is not justified enough. It is not shown that the inhibition of tannic acid is specific. Why did the authors use this inhibitor and not others? Also, the authors do not discuss possible off-target effects of this compound in the results or discussion.

**Response:** Thank you very much for your comment. In accordance with your suggestion, we have supplemented the Discussion section with a justification for the use of tannic acid as an inhibitor: "Previous studies have demonstrated that tannic acid has the strongest inhibitory effect on pyruvate kinase, both in enzyme activity assays and in vitro growth inhibition experiments. Moreover, compared with other inhibitors, tannic acid is less cytotoxic [48]. " With respect to the specificity of the inhibitory effect of tannic acid, we fully acknowledge your point that the current research has not yet established tannic acid as a specific inhibitor of pyruvate kinase. Accordingly, the term "specific" was inappropriate, and we have removed it from the revised manuscript. To address the potential off-target effects of tannic acid, we have added a dedicated discussion in the revised manuscript: "Notably, as a natural polyphenolic compound, tannic acid possesses multiple protein‒binding sites, suggesting the possibility of off‒target effects [51]. However, some studies have revealed relatively specific mechanisms of action. For instance, in cancer research, Yang et al. [52] reported that tannic acid directly and selectively binds to the K433 site of PKM2, inhibiting its activity without affecting that of PKM1 and thereby inducing apoptosis in cancer cells. Nevertheless, given the structural differences between parasite and mammalian proteins, the potential off‒target effects of tannic acid in parasites require further elucidation. "

An X, Yu L, Wang S, Ao Y, Zhan X, Liu Q, et al. Kinetic Characterization and Inhibitor Screening of Pyruvate Kinase I From Babesia microti. Front Microbiol. 2021; 12: 710678.

HK. Phytochemical innovations in oral cancer therapy: targeting oncogenic pathways with natural compounds. J Complement Integr Med. 2025 Jul 11.

Yang P , Ding GB , Liu W , Fu R , Sajid A , Li Z . Tannic acid directly targets pyruvate kinase isoenzyme M2 to attenuate colon cancer cell proliferation. Food Funct. 2018 Nov 14;9(11):5547-5559.

**Q5** About the “In vitro inhibition assay”:

As described, the “In vitro inhibition assay” section lacks sufficient biological validity to support the conclusions drawn. The data appear to be based on only two individual worms; one treated with 100 µM tannic acid and one untreated control. This very high inhibitor concentration is not justified in the text, nor is it discussed in the limitations section.

No biological replicates are described, making it impossible to assess reproducibility or variability. Without independent replicates, these results should be considered preliminary. The authors should repeat the experiment with an appropriate number of biological replicates and controls before drawing definitive conclusions.

Furthermore, it is unclear how statistical analyses and error bars were generated from the experiment as described. The manuscript should clearly state the number of biological and technical replicates, the statistical tests used, and the nature of the error bars. Without this information, the statistical validity of the findings cannot be evaluated.

**Response:** Thank you very much for your comment. With respect to the rationale for the inhibitor concentration, we have supplemented the Discussion section as follows: "Tannic acid inhibited r*Sm*PK1 enzymatic activity in a concentration-dependent manner, resulting in complete inhibition at 8 μM. An et al. [48] reported that 20 µM tannic acid treatment inhibited *Babesia microti* growth by 80% after 3 d of incubation, and the inhibitory effect was further enhanced when the concentration was increased to 100 µM. Yue et al. [24] reported that the enzymatic activity of native *Trichinella spiralis* PK in muscle larval proteins was reduced by 12.82, 20.45, 51.77 and 65.65% respectively under various doses of tannic acid (25, 50, 75 and 100 μM) treatment. Li et al. [32] reported that *Babesia duncani* growth was inhibited by nearly 100% under 10 µM tannic acid treatment, suggesting a potential link between PK inhibition and growth suppression."

Considering that *Spirometra mansoni* is significantly larger in size than *Trichinella spiralis* and *Babesia* are and considering our preliminary experimental data, we ultimately selected a maximum dose of 100 µM. Second, with respect to biological replicates, obtaining adult tapeworms is extremely challenging, as it requires the infection of a definitive host. Under laboratory conditions, the entire process demands stringent ethical approval. During our application for ethical clearance, after repeated communications, our university’s ethics committee permitted us to infect only one cat. Considering that a high infection dose could lead to intestinal obstruction, we ultimately infected the cat with 3 plerocercoids from the same host on the basis of previous studies. One worm was used for experiments such as qPCR and IFA, one was used for in vitro inhibition experiments, and the remaining worm served as a control. As you rightly pointed out, in the absence of independent replicates, these results should be considered preliminary. Therefore, we have also addressed the experimental limitations in the Discussion section: "Notably, this study preliminarily validated the functional role of pyruvate kinase in the growth and development of *Spirometra mansoni* through in vitro enzyme inhibition experiments. However, owing to the challenges in sample availability and the lack of a long‒term in vitro culture system for the parasite, this study did not employ more advanced genetic techniques (such as RNA interference or CRISPR/Cas9 gene editing) to perform loss‒of‒function experiments for direct validation of gene function. Once a stable culture system is established in the future, further targeted genetic experiments will be conducted to provide more critical evidence for target validation. "

Finally, regarding the issue of statistical testing methods, we have supplemented the number of technical replicates, the statistical test method used, and the nature of the error bars in the figure legends. : "Statistical analysis was performed using SPSS 26 software. Statistical significance was determined by one-way analysis of variance (ANOVA), with data derived from three technical replicates. A **p*-value < 0.05 was considered to indicate statistical significance. the error bars represent the means ± standard deviations."

Yue WW, Yan SW, Zhang R, Cheng YK, Liu RD, Long SR, et al. Characterization of a novel pyruvate kinase from Trichinella spiralis and its participation in sugar metabolism, larval molting and development. PLoS Negl Trop Dis. 2022; 16: e0010881.

An X, Yu L, Wang S, Ao Y, Zhan X, Liu Q, et al. Kinetic Characterization and Inhibitor Screening of Pyruvate Kinase I From *Babesia microti*. Front Microbiol. 2021; 12: 710678.

**Other points**

**Q6** Figure 1 – As I mentioned before “Medical Tapeworm” is not an appropriate sentence, and it is here as a title in the image.

**Response:** Thank you for reminding us of this issue again, and we sincerely apologize for not thoroughly addressing this expression in our previous revision. This was indeed an oversight on our part. In accordance with your comments, we have conducted a thorough and detailed check of the entire manuscript to ensure that the inappropriate term "Medical Tapeworm" has been completely revised.

**Q7** Line 160 – “Adult worms were obtained as previously described [26].” Please provide at least a short description of how this was carried out.

**Response:** Thank you for raising this question. We have supplemented the specific method for obtaining adult worms in the manuscript: "Adult worms were obtained as previously described [26]. More specifically, 3 plerocercoids from the same host were perorally administered to a 6-month-old specific pathogen free domestic cat. Forty days after the infection, the cat was preanaesthetized and then euthanized and the adult worms were recovered from the small intestine. "

**Q8** Line 163-164 – “Female Kunming mice, aged 4-6 weeks, were purchased from the Henan Laboratory Animal Center.” It is not in context why this is mentioned here.

**Response:** Thank you for your valuable comments. In accordance with your suggestions, we have revised the original text to clarify the specific purpose of using this strain of mouse and the subsequent procedures. We have revised the sentence "4–6-week-old female Kunming mice were purchased from Henan Experimental Animal Center" to "Moreover, to obtain related immune serum, 4–6-week-old female Kunming mice (purchased from Henan Experimental Animal Center) were used in this study for recombinant protein immunization. The obtained anti-recombinant immune serum was stored at -80 °C for later use.

**Q9** Line 168 – It is not clear if the “samples = 3” for SNIP, MP and GP, are from three different worms or are 3 different sections of the same worms divided in three.

**Response:** Thank you for raising this question. These samples were indeed obtained from three distinct tapeworms. We have revised the sentence as follows: "Total RNA was extracted using TRIzol reagent (Invitrogen, USA). For each proglottid type (SNIP, MP, and GP), three biological replicates were performed, with each replicate sample derived from the same individual tapeworm (totalling three worms). Consequently, three independent RNA samples were obtained for each group. "

**Q10** Figure 3 Part A – It is not clear what are the numbers in the figure.

**Response:** Thank you for your question. The numbers inside the boxes in the figure represent genes. Revised figure caption: "**A.** Fifteen DEGs involved in the glycolytic pathway were identified in the SNIPs and MPs. The numbers in boxes represent genes. Red symbols represent genes upregulated in MPs. Yellow symbols represent genes with mixed regulation."

**Q11** Figure 3 Part C – The format of the font is not uniform in the image.

**Response:** Thank you for pointing out the inconsistency in font formatting in Figure 3C. In the revised manuscript, we have modified Figure 3C as follows.

We are very grateful for your useful comments and suggestions on the language and structure of our manuscript. We have improved and made corrections in the revised manuscript. We earnestly appreciate the Editor’s/Reviewers’ warm remarks and hope that the revised manuscript will meet the requirements of this journal. Once again, thank you very much for your comments and suggestions.
